# Implementation of a Mindful Walking Intervention in Breast Cancer Patients After Their Primary Oncologic Treatment: Results of a Qualitative Study Within a Randomized Controlled Trial

**DOI:** 10.1177/15347354241237972

**Published:** 2024-04-23

**Authors:** Miriam Ortiz, Maren Luise Schröder, Benno Brinkhaus, Barbara Stöckigt

**Affiliations:** 1 Institute of Social Medicine, Epidemiology and Health Economics, Charité - Universitätsmedizin Berlin, Corporate Member of Freie Universität Berlin, Berlin, Germany

**Keywords:** breast cancer, mindfulness, walking, qualitative research, coping

## Abstract

**Background:**

Breast cancer survivors often suffer from diagnosis- and therapy-related long-term side effects, such as cancer related fatigue, restricted stress resilience and quality of life. Walking as a physical activity and mindfulness practice have been shown to be helpful in studies. The aim of this study was to compare the individual experiences and subjectively perceived effects of walking in combination with mindfulness practice with moderate walking alone in breast cancer patients. This paper focuses on the qualitative results of a mixed-methods pilot study.

**Methods:**

Breast cancer patients who had finished their primary oncologic treatment at least 6 months ago were randomized to an 8-week group intervention program of either mindful walking or moderate walking. Within the qualitative study part, semi-structured focus group interviews (2 interviews per study arm) were conducted and analyzed using a qualitative content analysis approach. Audio recorded interviews were transcribed verbatim and pseudonymized. The subsequent data analysis was performed by using MAXQDA^®^.

**Results:**

A total of 51 women (mean age 55.8 [SD 10.9] years) were included in the RCT, among these 20 (mean age 56.7 [SD 12.0] years) participated in the focus group interviews (n = 11 patients of the mindful walking group; n = 9 patients of the walking group). Breast cancer patients in both groups described different effects in the complex areas of self-efficacy, coping, body awareness and self-reflection. While mindful walking primarily promoted body awareness and inner strength by mindfulness in breast cancer patients, moderate walking promoted self-efficacy by a confidence of their body and an easily integrated and accepted way of physical activity.

**Conclusions:**

Study interventions and the study setting triggered processes and reflections on one’s own health and situation. However, mindful walking and moderate walking seem to address different resources. This important knowledge may help oncologists and other therapists to assess what type of interventions can best meet the needs and requirements of individual patients.

**Trial registration:**

DKRS00011521; prospectively registered 21.12.2016; https://drks.de/search/de/trial/DRKS00011521

## Background

Breast cancer (BC) diagnosis, therapy and survival are often associated with long-term physical and psychological consequences. Quality of life (QoL) may be restricted in role, emotional, cognitive and social functioning. Symptoms of insomnia, fatigue, and depression are often persisting in oncology patients with long-term survival.^1-3^ Possibly in this context about 50% of all cancer patients, especially BC patients, use integrative medicine within the course of theirdisease^4,5^. Integrative oncology has been defined as “patient-centered, evidence-informed field of cancer care that utilizes mind and body practices, natural products, and/or lifestyle modifications from different traditions alongside conventional cancer treatments. Integrative oncology aims to optimize health, quality of life, and clinical outcomes across the cancer care continuum and to empower people to prevent cancer and become active participants before, during, and beyond cancer treatment.”^6^ Physical activity and mindfulness-based interventions may be considered as part of a mind-body oriented lifestyle intervention in the frame of integrative oncology. The research results on walking as well as on mindfulness-based interventions in BC patients illustrate the effectiveness and relevance of both types of interventions in the treatment and long-term medical care of breast cancer (BC) patients.^7-10^

In order to explore possible combined effects of these interventions we developed a new study intervention comprising walking elements and mindfulness meditation (mindful walking [MFW]).

The aim of this study was to compare the individual experiences and subjectively perceived effects of MFW with moderate walking (MW) alone in breast cancer patients.^11^ Identifying differences in the mode of action of the 2 study groups could be of interest for clinical practice regarding recommendations for patients and further research. This article focuses solely on the qualitative results of the trial and describes them in detail.

## Methods

### Study Design and Ethics Approval

The qualitative study part was nested in a randomized controlled, 2-armed, mixed-methods pilot study, carried out at the Institute for Social Medicine, Epidemiology and Health Economics, Charité—Universitätsmedizin Berlin, Germany from 2016to 2018. The mixed-method results of the study were published elsewhere with a focus on the quantitative part.^11^

Qualitative research aims to describe, openly explore, and understand “from within,” meaning to include subjectivity, individuality, the situation, reflections and interactions.^12^ The qualitative study component was chosen to explore subjective and individual experiences and perceptions in more depth and to gain a more comprehensive understanding of the similarities and differences between the 2 intervention groups.

The study was approved by the ethics committee of the Charité—Universitätsmedizin Berlin (EA1/201/16; 06.07.2016) based on the Declaration of Helsinki and ICH E6 Guideline for Good Clinical Practice (GCP). Written informed consent was obtained from all individual participants included in the study. This article follows the Standards for Reporting Qualitative Research (SRQR) (Supplement 1).^13^

### Participants

Inclusion criteria were female BC patients, ≥18 years of age, who had completed their primary cancer therapy (operation, chemotherapy, radiation therapy) at least 6 months before the beginning of the study intervention. Further inclusion and exclusion criteria are presented in detail in the mixed-method paper.^11^ For the qualitative study component all participants who had attended at least 6 out of 8 course dates, were contacted after the follow-up time of 16 weeks or longer and invited to participate in the focus group interviews. We assumed that a minimum number of 6 attended course dates was relevant to evaluate the intervention appropriately. Participation in the focus groups was voluntary.

### Study Interventions

After randomization, participants of both groups, MFW and MW, attended a 90-minute group intervention once a week for 8 weeks. A maximum number of 10 participants per group was determined in advance as the maximum group size on the recommendation of the qualified and experienced trainers, as their experience has shown that courses of this type are then more feasible.

Participants of MFW performed mindfulness meditation exercises such as breathing meditation, Metta meditation (loving kindness) and body scan in combination with short walking sessions. Participants of MW only practiced moderate walking outside without mindfulness meditation exercises. Both groups were encouraged by their trainers to perform the respective intervention as a home practice. Further details are given elsewhere.^11^

### Qualitative Data Collection and Analysis

The qualitative data collection took place after the quantitative evaluation at a follow-up time of 16 weeks. A longer time period between the end of the follow-up time and the focus group interview came about, to bring together course participants from different MFW or MW courses in the interviews.

Two focus groups per study arm were planned in advance and conducted by M.S. and B.S., 2 experienced qualitative researchers and part of the research team. Focus groups were conducted because they represent a shared space of experience. Thus, according to Mangold “collective interactions” of the respective group intervention can be pointed out.^14^

**Figure 1.**
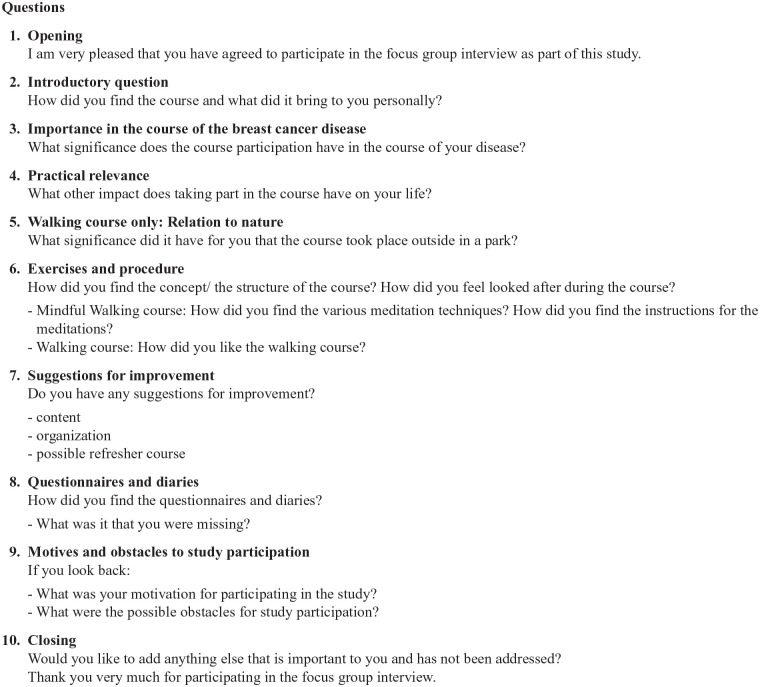
Interview guideline.

Prior to qualitative data collection the research team developed an interview guideline including the following topics (Figure 1—Interview Guideline):

- subjectively perceived experience of course participation
- significance of course participation in the frame of the BC disease
- subjectively perceived effects of course participation on everyday life
- feasibility of course participation

The focus group interviews were recorded digitally, transcribed verbatim and pseudonymized. The following data analysis was carried out deductively and inductively using a qualitative content analysis approach.^15^ Codes were created both deductively based on the interview guideline and inductively from text data material. Subsequently, the codes were discussed by the qualitative researchers and the research team and decided upon in a consent procedure. Furthermore, based on Grounded Theory, memo writing was used as an analytic element. Memos are notes that function as a description to help to describe, further develop, and reflect the codes throughout data analysis.^16^ The data analysis was performed using the computer program MAXQDA^®^.

Data analysis and its results were discussed regularly in addition in an interdisciplinary qualitative working group (Qualitative Research Network, Charité—Universitätsmedizin Berlin) to consider different perspectives and to ensure intersubjectivity and trustworthiness. Thus, the quality and validity of the qualitative data analysis was improved. The research team consisted of 4 physicians: 3 specialized in integrative medicine, 2 experienced in qualitative research, and 1 with a specialization in anthropology.

## Results

The study was conducted between September 2017 und April 2019. Fifty-one patients were included in the mixed-method study and randomized into groups (MFW n = 24; MW n = 27); 32 patients were contacted to take part in the focus group interviews after the follow-up period. On a voluntary basis, 20 patients agreed to participate in 4 focus group interviews, 2 focus group interviews per study arm; 12 patients declined participation in the focus group interviews mainly due to time constraints and management. The focus group interviews consisted of 4 to 6 participants each. All interviews took place at the Institute for Social Medicine, Epidemiology and Health Economics, Charité—Universitätsmedizin Berlin. The mean age of focus group participants was 56.7 years (SD 12.0).

The following codes emerged deductively and inductively from the focus group interviews: *evaluations and effects of course participation*, *doubts and criticism*, *reference to nature*, *suggestions for improvement*, *questionnaires*, *diaries*, *refresher course*, *study participation*, *the group*, *conventional medicine vs. integrative oncology*, *support groups*, *ambivalence* and *interaction and group atmosphere*. The main codes partly contained further subcodes in a second, third and fourth level.

**Figure 2.**
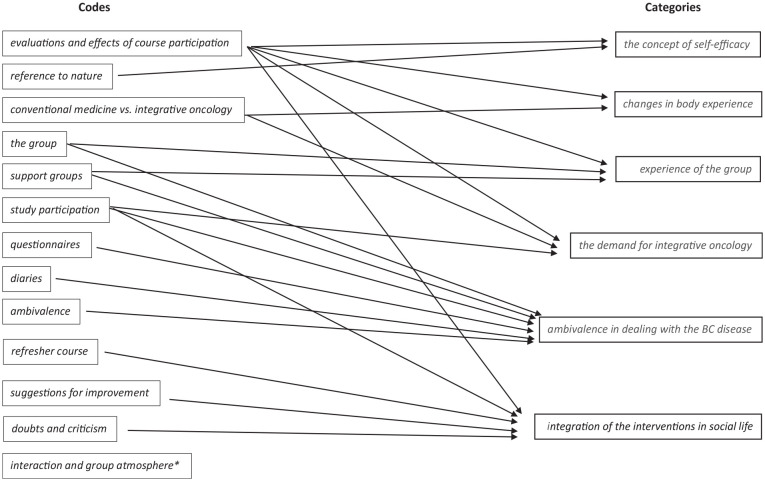
Codes and Categories. *This code refers to conspicuousness within the focus group interviews such as parts of speech, mood, intonation of what was said, atmosphere and non-verbal behavior. In addition, interaction between the participants are included under this code, for example, reference to each other, initiation and deepening of a topic in the interaction between the participants, weighting of a topic by interview participants. The codes are supplemented and completed by explanatory memos.

The following 6 central categories emerged from the relationships and interactions of the codes (Figure 2): *experience of the group*, *changes in body experience*, *integration of the interventions in social life*, *the concept of self-efficacy*, *ambivalence in dealing with the BC disease* and *the demand for integrative oncology*. In the following, these categories are to be explained in detail and compared between groups.

### Experience of the Group

The following aspects emerged regarding the group experience in both MFW and MW: Participants of both groups emphasized the importance of a group of women affected by BC, who could build on the commonality of the disease:

*“It is a protected space, but on the other hand it is also open for those who are inside, and you don’t have to explain yourself.” MFW_8, MindfulWalking_II*

However, there was a strong demarcation from existing support groups (see also *Ambivalence in Dealing with the BC Disease*). In distinction to support groups, participants of both groups emphasized the importance of the common task respectively walking and/or meditation within the framework of the group intervention.

The possibility of the exchange of experiences among BC patients along the course was positively emphasized by participants of MW.

*“You also get out [and] meet like-minded people [. . .] that is definitely important as well and one [can] get into a conversation with each other, exchange, and then do the same thing together.” MW_6, Walking_II*

### Changes in Body Experience

Throughout the study, participants of both groups described a change in their body experience, however MFW and MW differed in their effects. MFW participants described that they became more aware of their bodies. Participants of MFW learned to deal with their bodies, which may have changed externally and internally as part of their BC treatment, and to accept their bodies and their new physicality.

*“I just got a different body awareness [. . .].” MFW_9, MindfulWalking_II*

In contrast, participants of MW emphasized that active walking enabled them to experience their bodies positively again. The participants reported a feeling of *“positive exhaustion”* through physical activity, whereas the primary cancer treatment was often characterized by negative exhaustion.

*“After the first meeting, I also thought, “Wow, now I’m really exhausted.” But it was [. . .] a positive [. . .] exhaustion, [in] connection [. . .] with the disease one is always confronted with negative exhaustion, [. . .] chemotherapy [. . .] or radiation [. . .], it is always a negative exhaustion. And [that] was the experience of positive exhaustion [. . .]. And over time [. . .] [the] condition [. . .] also got better and better. [. . .] A [. . .] good experience, a good body experience [. . .], to notice [. . .] it’s getting easier and easier for me and [. . .] to experience the own body in a positive way again.” MW_7, Walking_II*

### Integration of the Interventions Into Social Life

Regarding the integration, feasibility, and acceptance of the interventions in the social environment and life, there were clear differences between MFW and MW.

MFW offered an opportunity to integrate a moment of “respite” in everyday life, to focus on and increase awareness of oneself, one’s surroundings and needs. However, at the same time, it was seen as difficult to integrate mindfulness into the everyday life and one’s (work) environment: the concept of mindfulness might be unrealistic in an achievement-oriented society and actions of self-care may provoke reactions of astonishment and incomprehension in the social environment. It was discussed that the expansion of personal space through the formulation of one’s own needs or limitations may allow new boundaries to emerge within a society, a group, or a family at the same time. Nevertheless, especially the increase of self-care was positively experienced by the participants.

*“I deliberately said, ‘No, I won’t do it, I’m reaching my limit here, I can’t do it anymore. [. . .] Someone else must do it.’ [. . .] I didn’t know that about myself [. . .], and [. . .] I noticed [. . .] that the environment had a lot of problems with it.” MFW_8, MindfulWalking_II*

MW, on the contrary, was reported to be easily integrated into the daily routine and seen as a socially accepted opportunity to do something good for oneself.

*“Since the beginning of March [. . .], I have been able to integrate [this] at my work [. . .] and now I go myself twice a week during working hours [. . .]. We do half an hour twice a week and it goes down well and I feel totally comfortable doing it [. . .].” MW_9, Walking_II*

However, it became apparent that after completion of the primary cancer treatment for both MW and MFW, even a fixed appointment once a week can be more difficult to integrate in one’s daily life due to the increased multiple burdens of work commitments, household, and family.

### Experience of Self-Efficacy

A central category and effect of both interventions was the promotion of self-efficacy, however generated in 2 different ways through MFW and MW.

MFW participants emphasized an *“inner strength,”* which was developed, trained, and promoted through mindfulness meditation as well as the strengthening of a conscious and self-determined way of dealing with oneself, one’s possibilities and limits. With the help of the MFW intervention, participants learned to take themselves more seriously and to focus on themselves and their own needs. This way of self-care led to an increase in self-efficacy.

*“I can deal better with my fears, which are always there, because I do these exercises and this meditation and that helps me [. . .], I simply feel stronger. [. . .] So even if I sometimes feel incredibly weak physically, I feel strong inside. [. . .] [And] I used to work for others [. . .] and always did several jobs and picked up the children and did this and that and at some point you clearly say stop. It’s also about self-love, you really have to take care of yourself [. . .] and this course has strengthened [that], [. . .]. I call it healthy egoism, [. . .] if I’m not doing well, it’s not good for anyone [. . .] and I clearly need these time-outs for myself.” MFW_9, MindfulWalking_II*

In contrast, MW participants described an increase in confidence of their physical performance through MW (see *Changes in Body Experience*). Self-efficacy is strengthened through MW itself, because it offers a means to care for oneself, as well as the positiveness of doing something good for oneself and one’s health.

*“Yes, [. . .] I don’t know, there is [. . .] this concept [of] self-efficacy [. . .] and [walking] is such a method, with which you can very quickly [. . .] do something good for yourself and [. . .] pat yourself on the back and say, ‘you’ve done well now’ [. . .].” MW_7, Walking_II*

The positive experience of nature was taken up by both intervention groups. MFW participants emphasized the increased attention and perception of nature as something beautiful. The conscious perception of nature in everyday life was seen as a way to be mindful. MW participants, however, felt that moving in nature was directly relaxing. Movement in nature allowed them to take time out from everyday life.

### Ambivalence in Dealing With the BC Disease

The statements made by participants of both groups, MFW and MW, revealed a certain ambivalence in dealing with their BC disease. Participants described a need to pick up the topic of their BC disease and its consequences again and to continue dealing with it. Nonetheless, participants also depicted a desire to leave the BC disease behind and to allow oneself to be healthy again. This inner conflict and ambivalence were reflected in various points: recognition and criticism of the questionnaires; the emphasis on the importance of having a group of affected BC patients versus the strong criticism of BC support groups; as well as motives and possible obstacles to study participation.

*“One of my considerations was, am I still a BC patient at all [. . .]? On the one hand [. . .], I found it quite pleasant [. . .] to tie in again and on the other hand [. . .] I also ask myself, [. . .] is it counterproductive to relate to it again, to identify myself as a BC patient, or do I just want to be healthy now?” MW_2, Walking_I*

### Demand for Integrative Oncology

Participants of both groups confirmed the importance of complementary medical treatment offers for BC patients during and/or after primary cancer therapy. Using complementary medicine, patients would regain a sense of control and self-efficacy, which they had often given up on and missed in the context of primary cancer therapy. Whereas in the standardized post-primary oncologic care setting, individual wishes and needs often remained disregarded, they were perceived and considered during both interventions. A desire for a more bio-psycho-social treatment approach especially in the aftercare of BC patients was evident in both groups. As a result, there was a demand for more integrative medicine offers in the context of cancer treatment and aftercare and, accordingly, an expansion of IO.

*“It’s nice [. . .] that conventional medicine is now opening a little to the subject of holism [. . .] that this is generally offered to women during a cancer illness [. . .] or afterwards [. . .] [and] that it is recognized, that we consist of body, mind and soul and not only of body [. . .] and that one does not only do chemo and radiation.” MW_9, Mindful Walking_II*

## Discussion

### Summary

The results of the qualitative study show the influence of both study interventions on illness, experience and coping, body perception regarding awareness and physical exercise, and self-care and self-efficacy. The study interventions and the study setting obviously triggered processes and reflections on one’s own health and situation although this is of course also influenced by individual aspects such as socio-demographic and clinical characteristics like the time since completion of the initial cancer treatment.^11^

### Strength and Limitations

This was the first study to our knowledge comparing different types of walking with a nested qualitative study that included several focus groups in the 2 different intervention groups. Both study interventions were characterized by a high relevance to everyday life: The mindful walking intervention followed an elaborate concept developed by an expert team. The concept was proofed in advance of the study regarding feasibility. Both walking interventions were easy to apply. Conducting focus group interviews provided the opportunity to elaborate collective experiences and thus come to a common consensus instead of focusing on individual experiences.

A limitation might be the sample of interviewed women: The interviewees had to have participated in at least 6 from 8 group dates, the participants who rarely came to the intervention dates or dropped out were not represented in the sample, thus a participation bias in the focus groups must be considered. In addition, the dynamic of focus groups may discourage certain participants from expressing opinions and experiences that differ from most of the other participants. Using other qualitative data collections methods, such as participatory observation of the study interventions, might have resulted in other insights.^17^ Another limitation might have been the varying length of the individual time between the end of the follow-up time point and the interview participation, that could have led to a recall bias. This happened because we wanted to mix participants from different courses of one intervention group. We decided for this model to avoid established group dynamics, but this also removed the familiar framework of an already known group, which could have led to more openness in the discussion.

### Discussion

The following sections discuss the results of the main categories identified in the qualitative analysis.

### The Role of the Group Setting

The group setting of the interventions created room for reflecting one’s own situation as a member of a cancer patient group with special needs. A different acceptance and legitimation for the formulation of one’s own health care needs may easily arise in a group of similarly affected persons in contrast to other social contexts. The reflections in the study groups during the practice of the different interventions may have enabled an increased exchange between the patients but it was not forced. By creating a safe and *“protected”* space for sharing, the group setting could have contributed to the deepening and strengthening of healing therapy effects through the general effective factors described by Spiegel et al, such as shared universal suffering, mutual instilling of hope and the exchange of information, and can thus also lead to improved acceptance and coping.^18^

### Body Experience During Interventions

Altered body image was inductively addressed by participants in both groups during focus group interviews: MFW promoted acceptance and a conscious approach to oneself and one’s own body, whereas MW activated participants and walking as such was perceived as a positive body experience. While the walking was immediately physically noticeable as outdoor exercise with subsequent perceived physical fatigue, the mindful walking rather triggered an intensified reflection process on the mental and spiritual level. The positive experiences go in line with the results of a systematic review, where Paterson et al highlight the importance of body image disturbances for young BC survivors and emphasize the need for further randomized intervention trials to assess and treat body image disturbances in BC survivors.^19^ Also, a further meta-analyses of 56 trials with 4826 patients have shown possible positive effects of physical activity among other on body image in cancer survivors.^8^

### Integration of Interventions in Social Life

Qualitative statements about the multiple burdens of professional work, household, and family of participants in both groups, as well as the time commitment as the main reason for nonparticipation in the study, dropouts, and noncompliance during the study, highlight barriers and difficulties of the interventions and are consistent with statements in the literature.^20^ Other studies show that the implementation of regular activities such as physical activity despite knowing about the benefits can be limited for BC patients by various, also gender-specific individual (eg, feeling guilty for taking care of yourself) or socio-cultural demands.^21,22^

Multiple stresses due to professional work and care work particularly affects women, highlighting existing gender inequalities in studies of female BC patients.^23^ Whereas MW was easy to integrate into everyday life and was socially accepted, MFW participants sometimes also reported resistance in the social environment and difficulties in applying individual mindfulness meditations.

Difficulties with individual mindfulness exercises and the large time commitment required to practice at home were also evident in a mixed-methods study by Eyles et al, who tested the feasibility of an MBSR intervention in patients with metastasized BC.^20^

### Self-efficacy

The disease itself, self-reflection, and training of self-care skills by interventions such as in our study leads often to a different level of self-care capabilities and self-efficacy.^24^ Self-efficacy, coined by Albert Bandura, can be defined as a person’s belief to successfully master challenges on their own and influence events that affect one’s life even in difficult situations.^25^

The strong desire for self-help and active participation as a motivation for study participation as well as the development of self-care and self-efficacy through the study interventions emerged as a central aspect in the focus group interviews of both groups. These findings are comparable to results on the use of integrative medicine found in the literature. A cross-sectional study by Theuser et al showed that the need to become active oneself can be fulfilled and coping strategies improved through the use of CIM.^26^ Hack et al showed that BC patients benefit from active participation and improved self-care through CIM.^27^ A meta-analysis by Merluzzi et al emphasized the relevance of self-efficacy as a positive resource for BC patients and as a target parameter for future studies.^28^

### Coping and Ambivalence

Health resources mentioned by our participants like feeling physically active (MW) and feeling connected to others and to their own fate (MFW) may not only lead to a short-term increase in well-being and the feeling of creating a room for self-care as a basis for self-efficacy, but also to a long- term improved coping with the underlying disease.^29^ Coping in the sense of Lazarus and Folkman helps to manage threatening situations by activating adequate resources, appraise the situation and adapt to it. Effective coping strategies include an optimistic approach, a self-confident approach and seeking social support. Ineffective coping methods include hopelessness and submissive approaches. Better coping using psychosocial interventions may influence long term survival in BC patients.^30^ A study of Stöckigt et al identified keeping a positive attitude toward life, choosing social contacts, and staying active as much as possible as crucial coping strategies in BC patients and found it related to quality of life.^29^ Further, in our study, nature as a space in which an intervention (MW) takes place and as a reference for a targeted perception training in the context of mindfulness exercises (MFW) was seen by our patients as a time-out in nature from everyday life and source for well-being, relaxation and enjoyment. That these aspects contribute to coping with cancer seems plausible. Various systematic reviews in which interventions also study different nature-based therapies support this assumption. A Swedish cross-sectional study found nature to be the most important coping factor for cancer patients.^31-33^

However, to deal with a chronic and possibly life-threatening disease often includes defense in the form of ambivalence in facing the illness.^34^ Especially “cancer-free” declared cancer survivors, like those in our study, often wish to leave the disease behind and focus unconditionally on their hard- won health. According to Kübler Ross’ model of coping in severe illness, defense against the diagnosis may be an important phase that can end with acceptance of the illness, which can open up new scope for action.^35^ Mindfulness training programs with reflecting tasks can be emotionally very confrontational and thus enhance ambivalence for patients.^36,37^ Bisseling et al found that the right time to participate in a mindfulness-based stress reduction program for BC patients depends more on their emotional readiness to let themselves feel vulnerable than on the stage of treatment.^38^ This point should be considered in counseling patients.

### Demand for Integrative Oncology

Especially for cancer patients, who often experienced feelings of powerlessness and loss of control due to the disease and its medical treatments, the feeling of self-efficacy and having influence on their own health remains limited sometimes.^39^ In this respect, health promotion interventions like in our study may empower patients by activating already known resources, such as walking, or opening up possible new resources, such as meditation. In this way, health-promoting courses from the field of physical activity and mindfulness often function as a kind of vehicle for self-efficacy in fighting or dealing with one’s own disease. Cancer patients acknowledge this and demand these interventions as well as others from the field of integrative oncology.^40^ Several guidelines for patients with BC or other cancer highlight the role of physical activity and mindfulness interventions.^41,42^ In Germany, however, courses in the field of movement therapy and mindfulness, such as MBSR courses, are often only subsidized as prevention courses for healthy individuals by the statutory health insurance.^43^ In the context of adequate patient care, this point should be changed, especially for chronically ill patients.

## Conclusion

Qualitative results of this exploratory study showed that breast cancer patients in both groups described effects of the study interventions regarding the complex areas of self-efficacy, coping, body awareness and self-reflection. In addition, the group setting of the study interventions seems to play a positive role as a framework to promote these aspects. Our main finding was that mindful walking and moderate walking seem to address different resources. While participants of MFW emphasized body awareness and inner strength by mindfulness, patients from MW experienced self-efficacy by a confidence of their body and an easily integrated and accepted way of physical activity. These insights may help oncologists and other therapists to assess what kind of interventions may meet individual patients′ needs and demands. As a result of the predominantly positive and inspiring experiences of the study patients we think that there is a high demand for more mind-body oriented offers from the field of integrative oncology in cancer patients.

## Supplemental Material

sj-docx-1-ict-10.1177_15347354241237972 – Supplemental material for Implementation of a Mindful Walking Intervention in Breast Cancer Patients After Their Primary Oncologic Treatment: Results of a Qualitative Study Within a Randomized Controlled Trial Supplemental material, sj-docx-1-ict-10.1177_15347354241237972 for Implementation of a Mindful Walking Intervention in Breast Cancer Patients After Their Primary Oncologic Treatment: Results of a Qualitative Study Within a Randomized Controlled Trial by Miriam Ortiz, Maren Luise Schröder, Benno Brinkhaus and Barbara Stöckigt in Integrative Cancer Therapies

## References

[1] Arndt V Merx H Stegmaier C Ziegler H Brenner H. Persistence of restrictions in quality of life from the first to the third year after diagnosis in women with breast cancer. J Clin Oncol. 2005;23:4945-4953. 16051947 10.1200/JCO.2005.03.475

[bibr2-15347354241237972] Jones SMW LaCroix AZ Li W , et al. Depression and quality of life before and after breast cancer diagnosis in older women from the Women’s Health Initiative. J Cancer Surviv. 2015;9:620-629. 25708515 10.1007/s11764-015-0438-y PMC4547920

[3] Mokhtari-Hessari P Montazeri A. Health-related quality of life in breast cancer patients: review of reviews from 2008 to 2018. Health Qual Life Outcomes. 2020;18:338. 33046106 10.1186/s12955-020-01591-x PMC7552560

[4] Horneber M Bueschel G Dennert G , et al. How many cancer patients use complementary and alternative medicine: a systematic review and metaanalysis. Integr Cancer Ther. 2012;11:187-203. 22019489 10.1177/1534735411423920

[5] Fremd C Hack CC Schneeweiss A , et al. Use of complementary and integrative medicine among German breast cancer patients: predictors and implications for patient care within the PRAEGNANT study network. Arch Gynecol Obstet. 2017;295:1239-1245. 28331996 10.1007/s00404-017-4348-2

[6] Witt CM Balneaves LG Cardoso MJ , et al. A comprehensive definition for integrative oncology. Natl Cancer Inst Monogr. 2017;2017:3-8. 10.1093/jncimonographs/lgx012 29140493

[7] Yuan Y Lin L Zhang N , et al. Effects of home-based walking on cancer-related fatigue in patients with breast cancer: a meta-analysis of randomized controlled trials. Arch Phys Med Rehabil. 2022;103:342-352. 34302791 10.1016/j.apmr.2021.06.020

[bibr8-15347354241237972] Mishra SI Scherer RW Snyder C , et al. Exercise interventions on health-related quality of life for people with cancer during active treatment. Cochrane Database Syst Rev. 2012;2012:Cd008465. 10.1002/14651858.CD008465.pub2 PMC7389071 22895974

[bibr9-15347354241237972] Schell LK Monsef I Wöckel A Skoetz N. Mindfulness-based stress reduction for women diagnosed with breast cancer. Cochrane Database Syst Rev. 2019;3:CD011518. 10.1002/14651858.CD011518.pub2 PMC6436161 30916356

[10] Ludwig DS Kabat-Zinn J. Mindfulness in medicine. JAMA. 2008;300:1350-1352. 18799450 10.1001/jama.300.11.1350

[11] Schröder ML Stöckigt B Binting S , et al. Feasibility and possible effects of mindful walking and moderate walking in breast cancer survivors: A randomized controlled pilot study with a nested qualitative study part. Integr Cancer Ther. 2022;21:1-13. 10.1177/15347354211066067 PMC8777370 35045736

[12] Flick U. Was ist qualitative Forschung? Einleitung und Überblick Qualitative Forschung: Ein Handbuch. 2003;11:13-29, Quotation page 14.

[13] O'Brien BC Harris IB Beckman TJ Reed DA Cook DA . Standards for reporting qualitative research: a synthesis of recommendations. Acad Med. 2014;89:1245-1251. 24979285 10.1097/ACM.0000000000000388

[14] M W . Gegenstand und Methode Des Gruppendiskussionsverfahrens. Europäische Verlagsanstalt; 1960.

[15] Hsieh HF Shannon SE. Three approaches to qualitative content analysis. Qual Health Res. 2005;15:1277-1288. 16204405 10.1177/1049732305276687

[16] C K . (ed.) Constructing Grounded Theory, 2nd ed. Sage; 2014.

[17] Schöne H. Die teilnehmende Beobachtung als Datenerhebungsmethode in der Politikwissenschaft. Methodologische reflexion und Werkstattbericht. FQS Forum Qual Soc Res. 2003;4:168-199.

[18] Spiegel D Bloom JR Yalom I. Group support for patients with metastatic cancer. A randomized outcome study. Arch Gen Psychiatry. 1981;38:527-533. 7235853 10.1001/archpsyc.1980.01780300039004

[19] Paterson CL Lengacher CA Donovan KA Kip KE Tofthagen CS. Body image in younger breast cancer survivors: a systematic review. Cancer Nurs. 2016;39:E39-E58. 10.1097/NCC.0000000000000251 PMC4607543 25881807

[20] Eyles C Leydon GM Hoffman CJ , et al. Mindfulness for the self-management of fatigue, anxiety, and depression in women with metastatic breast cancer: a mixed methods feasibility study. Integr Cancer Ther. 2015;14:42-56. 25161198 10.1177/1534735414546567 PMC4390604

[21] Sander AP Wilson J Izzo N Mountford SA Hayes KW. Factors that affect decisions about physical activity and exercise in survivors of breast cancer: a qualitative study. Phys Ther. 2012;92:525-536. 22156026 10.2522/ptj.20110115

[22] Sequeira M Luz R Alvarez MJ. The practice of physical activity after breast cancer treatments: a qualitative study among Portuguese women. Front Psychol. 2022;13:1-11. 10.3389/fpsyg.2022.823139 PMC8965006 35369245

[23] McDonough MH Beselt LJ Kronlund LJ , et al. Social support and physical activity for cancer survivors: a qualitative review and meta-study. J Cancer Surviv. 2021;15:713-728. 33128705 10.1007/s11764-020-00963-y

[24] Foster C Breckons M Cotterell P , et al. Cancer survivors’ self-efficacy to self-manage in the year following primary treatment. J Cancer Surviv. 2015;9:11-19. 25028218 10.1007/s11764-014-0384-0 PMC4341005

[25] B A . Self Efficacy. Academic Press; 1994:71-81.

[26] Theuser AK Antoniadis S Langemann H , et al. Active participation, mind–body stabilization, and coping strategies with integrative medicine in breast cancer patients. Integr Cancer Ther. 2021;20:1-11. 10.1177/1534735421990108 PMC7924003 33645304

[27] Hack CC Antoniadis S Hackl J , et al. Breast cancer patients’ satisfaction with individual therapy goals and treatment in a standardized integrative medicine consultancy service. Arch Gynecol Obstet. 2018;298:147-156. 29704060 10.1007/s00404-018-4779-4

[28] Merluzzi TV Pustejovsky JE Philip EJ , et al. Interventions to enhance self-efficacy in cancer patients: a meta-analysis of randomized controlled trials. Psychooncology. 2019;28:1781-1790. 31206917 10.1002/pon.5148 PMC6731146

[29] Stöckigt DMB Kirschbaum B Carstensen DMM Witt DMCM Brinkhaus DMB . Prophylactic acupuncture treatment during chemotherapy in patients with breast cancer – results of a qualitative study nested in a randomized pragmatic trial. Integr Cancer Ther. 2021;20:1-10. 10.1177/15347354211058207 PMC8646188 34814766

[30] Stagl JM Lechner SC Carver CS , et al. A randomized controlled trial of cognitive-behavioral stress management in breast cancer: survival and recurrence at 11-year follow-up. Breast Cancer Res Treat. 2015;154:319-328. 26518021 10.1007/s10549-015-3626-6 PMC5752103

[31] Ray H Jakubec SL. Nature-based experiences and health of cancer survivors. Complement Ther Clin Pract. 2014;20:188-192. 25160991 10.1016/j.ctcp.2014.07.005

[bibr32-15347354241237972] Timko Olson ER Olson AA Driscoll M Vermeesch AL. Nature-based interventions and exposure among cancer survivors: a scoping review. Int J Environ Res Public Health. 2023;20:1-12. 10.3390/ijerph20032376 PMC9916332 36767741

[33] Ahmadi F Ahmadi N. Nature as the most important coping strategy among cancer patients: a Swedish survey. J Relig Health. 2015;54:1177-1190. 24363200 10.1007/s10943-013-9810-2 PMC4461799

[34] Lazarus RS Folkman S. Stress, Appraisal and Coping. Springer; 1984.

[35] Kübler-Ross E. On Death and Dying. Routledge; 1973.

[36] Schellekens MP van den Hurk DG Jansen ET , et al. Perspectives of bereaved partners of lung cancer patients on the role of mindfulness in dying and grieving: a qualitative study. Palliat Med. 2021;35:200-208. 33308039 10.1177/0269216320967281

[37] Krebber AH van Uden-Kraan CF Melissant HC , et al. A guided self-help intervention targeting psychological distress among head and neck cancer and lung cancer patients: motivation to start, experiences and perceived outcomes. Support Care Cancer. 2017;25:127-135. 27585808 10.1007/s00520-016-3393-x PMC5127860

[38] Bisseling EM Schellekens MPJ Jansen ETM , et al. Mindfulness-based stress reduction for breast cancer patients: a mixed method study on what patients experience as a suitable stage to participate. Support Care Cancer. 2017;25:3067-3074. 28470371 10.1007/s00520-017-3714-8 PMC5577047

[39] Chirico A Lucidi F Merluzzi T , et al. A meta-analytic review of the relationship of cancer coping self-efficacy with distress and quality of life. Oncotarget. 2017;8:36800-36811. 28404938 10.18632/oncotarget.15758 PMC5482699

[40] Greenlee H Neugut AI Falci L , et al. Association between complementary and alternative medicine use and breast cancer chemotherapy initiation: the breast cancer quality of care (BQUAL) study. JAMA Oncol. 2016;2:1170-1176. 27243607 10.1001/jamaoncol.2016.0685 PMC5484521

[41] Greenlee H DuPont-Reyes MJ Balneaves LG , et al. Clinical practice guidelines on the evidence-based use of integrative therapies during and after breast cancer treatment. CA Cancer J Clin. 2017;67:194-232. 28436999 10.3322/caac.21397 PMC5892208

[42] S3-Leitlinie Komplementärmedizin in der Behandlung von onkologischen PatientInnen: Leitlinienprogramm Onkologie der Arbeitsgemeinschaft der Wissenschaftlichen Medizinischen Fachgesellschaften e.V. (AWMF), Deutschen Krebsgesellschaft e.V. (DKG) und Deutschen Krebshilfe (DKH);2021. Accessed December 2, 2023. https://www.leitlinienprogramm-onkologie.de/fileadmin/user_upload/Downloads/Leitlinien/Komplement%C3%A4r/Version_1/LL_Komplement%C3%A4r_Langversion_1.1.pdf.

[43] Spitzenverband G . Leitfaden Prävention. 2024. Accessed March 12, 2024. https://www.gkv-spitzenverband.de/krankenversicherung/praevention_selbsthilfe_beratung/praevention_und_bgf/leitfaden_praevention/leitfaden_praevention.jsp.

